# Reactive Oxygen Species Detection Using Fluorescence in *Enchytraeus crypticus*—Method Implementation through Ag NM300K Case Study

**DOI:** 10.3390/toxics9100232

**Published:** 2021-09-24

**Authors:** Susana I. L. Gomes, Ana B. Neves, Janeck J. Scott-Fordsmand, Mónica J. B. Amorim

**Affiliations:** 1 Department of Biology & CESAM, University of Aveiro, 3810-193 Aveiro, Portugal; anab.neves@ua.pt (A.B.N.); mjamorim@ua.pt (M.J.B.A.); 2 Department of Bioscience, Aarhus University, Vejlsovej 25, P.O. Box 314, DK-8600 Silkeborg, Denmark; jsf@bios.au.dk

**Keywords:** oxidative stress, DCFH-DA method, fluorescence microscopy, soil invertebrates, nanomaterials

## Abstract

An imbalance between reactive oxygen species (ROS) and antioxidants in a living organism results in oxidative stress. Measures of such imbalance can be used as a biomarker of stress in ecotoxicology. In this study, we implemented the ROS detection method based on the oxidant-sensing probe dichloro-dihydro-fluorescein diacetate (DCFH-DA), detected by fluorescence microscopy, in *Enchytraeus crypticus* adults and cocoons, i.e., also covering the embryo stage. Hydrogen peroxide (H_2_O_2_), a well-known ROS inducer, was used both to optimize the method and as positive control. Implementation was successful, and the method was used to assess ROS formation in *E. crypticus* cocoons and adults when exposed to the reference silver nanomaterial Ag NM300K, at two effect concentrations (EC20 and EC50) for both hatching and reproduction over 3 and 7 days. The measured ROS levels varied with time, concentration, and developmental stage, with higher levels detected in adults compared with cocoons. In cocoons, ROS levels were higher at the EC20 than the EC50, which could be explained by non-monotonic concentration-response curve for hatching and reproduction, as previously observed. The increase in ROS levels at day 3 preceded the oxidative damage, as reported to occur later (day 7) in adults. The DCFH-DA method was successfully implemented here and can be further used as a new tool to detect ROS formation in *E. crypticus*, especially after short-term exposure to chemicals, including nanomaterials. We recommend the use of 3 and 7 days in the exposure design for this assessment.

## 1. Introduction

Oxidative stress biomarkers are often used in ecotoxicology for the evaluation of environmental quality, as well as to investigate the toxicity mechanisms of many common xenobiotics [1]. Several methods to evaluate biomarkers of oxidative stress (e.g., superoxide dismutase (SOD), catalase (CAT), glutathione peroxidases (GPx), glutathione reductase (GR), glutathione-S-transferase (GST), glutathione levels (GSH), metallothionein (MT), and so on) and damage (e.g., lipid peroxidation (LPO) and lactate dehydrogenase (LDH)) are available and routinely applied in soil ecotoxicology model species. Examples include earthworms (e.g., [2,3,4,5]), enchytraeids (e.g., [6,7,8,9]), and collembolans (e.g., [10,11]). However, methods to detect the formation of reactive oxygen species (ROS), instead of the defence mechanisms they activate (SOD, CAT, GPx, and so on), or the damage they cause (LPO), are far less often used in soil ecotoxicology model species (the few examples include [12,13,14]).

The detection of ROS based on the 2′,7′-dichlorodihydrofluorescein diacetate (DCFH-DA) method (DCFH is produced after hydrolysis of DCFH-DA in the cell, with this being oxidized by ROS into a fluorescent product, and hence detected, e.g., by fluorescence microscopy) is routinely used in cell lines, e.g., to assess the effects of nanomaterials (NMs) (e.g., [15,16,17]). The same method has been implemented in multicellular whole organisms, namely *Folsomia candida* (applied to dead animals [12]) and *Caenorhabditis elegans* (applied to live animals [13]). This procedure allows the measurement without tissue homogenization, as required for the above mentioned biomarkers of oxidative stress and damage. Hence, this approach in principle has an advantage owing to the possibility of being able to visualize the whole animal.

In the present study, we aimed to implement the procedures for ROS detection in *Enchytraeus crypticus* (adults and cocoons), using the DCFH-DA method and fluorescence microscopy. This was based on the methodology previously reported for *F. candida* (on dead animals [12]). *E. crypticus* (Oligochaeta) is a model species in soil ecotoxicology [18], with a vast array of other endpoints available, including molecular [19] and oxidative stress and damage cell level endpoints [8,20]. The method implementation included exposure to H_2_O_2_, a known ROS inducer. After the successful implementation, the impact of a silver (Ag) nanomaterial (NM) (the JRC standard Ag NM300K) was assessed, in adults and cocoons, exposed to the effect concentrations that induce 20 and 50% reduction on reproduction and hatching (EC20 and EC50) [21], over 3 and 7 days. Ag NM was used because it is among the most used NMs worldwide and they are known to be toxic to many non-target organisms, e.g., plants [22] and soil invertebrates, such as *E. crypticus* [8], *E. albidus* [7], *Eisenia fetida* [4], and *F. candida* [10]. Further, in *E. crypticus*, the effects of the Ag NM300K have been thoroughly assessed at organism [21] gene level [19], oxidative stress biomarkers [8], and DNA damage [20], thus providing a consolidated knowledge frame for the interpretation of the results obtained.

## 2. Materials and Methods

### 2.1. Test Species

*Enchytraeus crypticus* [23] was used as test species. Cultures were kept in agar plates, consisting of sterilized Bacti-Agar medium (Oxoid, Agar No. 1) and a mixture of four different salt solutions at the final concentrations of 2 mM CaCl_2_·2H_2_O, 1 mM MgSO_4_, 0.08 mM KCl, and 0.75 mM NaHCO_3_, under controlled conditions of temperature (19 ± 1 °C) and photoperiod (16:8 h light/dark). The cultures were fed with ground autoclaved oats twice per week. Synchronized age cocoons (1–2 days post-laying) and mature adult organisms were used. For further details on cultures’ synchronization, please see Bicho et al. [24].

### 2.2. Test Soil

The natural standard LUFA 2.2 soil (Speyer, Germany) was used. Its main characteristics are as follows: pH (0.01 M CaCl_2_) = 5.6; organic carbon = 1.61%; cation exchange capacity (CEC) = 8.5 meq/100 g; maximum water holding capacity (maxWHC) = 43.3%; and a grain size distribution of 8.9% clay (<0.002 mm), 13.9% silt (0.002–0.05 mm), and 77.2% sand (0.05–2.0 mm). The soil was dried (48 h, 60 °C) before use.

### 2.3. Test Materials, Characterization, and Spiking Procedures

2′,7′-Dichlorofluorescin diacetate (DCFH-DA) (Sigma-Aldrich Chemicals Co. (St. Louis, MO, USA)) was used as the fluorometric probe for detection of ROS. Hydrogen peroxide (H_2_O_2_) solution (35%, Merck) was used as oxidative stress inducer and as positive control.

The reference Ag nanomaterial (Ag NM300K) from the European Commission Joint Research Centre, fully characterized [25], was used as test material because it is also known to cause oxidative stress to *E. crypticus* [8]. In short, Ag NM300K particles are spherical and consist of a colloidal dispersion with a nominal Ag content of 10.2% *w*/*w*, dispersed in 4% *w*/*w* of polyoxyethylene glycerol trioleate and polyoxyethylene (20) sorbitan monolaurate (Tween 20), having >99% of particles with a nominal size of approximately 15 nm, with no coating. Transmission electron microscopy (TEM) indicated a size of 17 ± 8 nm. Smaller nanoparticles of approximately 5 nm are also present. The dispersant was also tested alone (control-dispersant).

The test concentrations of Ag NM300K were selected based on known reproduction and hatching effect concentrations (EC20 and EC50) [21]. The details and test concentrations, for H_2_O_2_ and Ag NM300K, are summarized in Table 1.

H_2_O_2_ was added to pre-moistened LUFA 2.2 soil, as serially diluted aqueous solutions, by test condition (concentration). The soil was thoroughly mixed, and water was added until 50% of soil maxWHC. The soil was mixed again and divided into each test vessel.

Ag NM300K (and the dispersant alone) were added to pre-moistened LUFA 2.2 soil, as serially diluted aqueous dispersions, replicate by replicate (to ensure raw amounts). The soil was thoroughly mixed, and water was added until 50% of soil maxWHC. The soil was left to equilibrate for 24 h prior to the test start.

Four replicates per test condition were performed, for both cocoons and adults. Each replicate consisted of 10 and 5 g of moist soil for adults and cocoons, respectively. For adults, 10 organisms with a well-developed clitellum were introduced in each test vessel (⌀ 4 cm glass vessel), corresponding to one replicate. For cocoons, five cocoons (1–2 days old post-laying, synchronized age) were introduced into each well of six-well plates (35mm ø), corresponding to one replicate. The tests ran for 3 and 7 days at 20 ± 1 °C with a photoperiod of 16:8 h (light/dark).

### 2.4. ROS Formation Assessment (Fluorescence Detection)

The organisms (adults and cocoons) of each replicate were collected, washed twice with MilliQ water, followed by another wash with 1× phosphate buffer saline (PBS, Sigma-Aldrich, Germany), and then incubated with 50 μM DCFH-DA in 1× PBS for 30 min while protected from light. After that, adults and cocoons were washed twice with 1× PBS buffer and placed onto a slide for microscopic examination. The fluorescence was detected at a wavelength of 535 nm, after the excitation of the material at 485 nm, using the microscope ZEISS Axioscope 5 (Oberkochen, Germany) with the Filter Set 38 HE (Oberkochen, Germany). The fluorescence microscope images were obtained using the camera ZEISS Axiocam 202 mono (Oberkochen, Germany), using the same exposure time of 20 ms per sample, and collected using the software Zen 3.1 Blue Edition. At least three organisms per replicate were photographed. The corrected total fluorescence (CTCF) was calculated, using the Image J software [26], applying the following formula (https://theolb.readthedocs.io/en/latest/imaging/measuring-cell-fluorescence-using-imagej.html) (accessed on 12 November 2020):

CTCF = Integrated Density − (Area of selected cell/organism × Mean fluorescence of background readings)

The results are expressed as arbitrary units.

### 2.5. Data Analysis

For both tests, (1) implementation and (2) case study, two-way analysis of variance (ANOVA) was used, with the Holm–Sidak test (*p* < 0.05) for all pairwise comparisons. CTCF was the dependent variable, and the independent variables were time (3 and 7 days) and test condition (test 1: 0, 500, 1000, 2000 mg H_2_O_2_/kg; test 2: control, control-dispersant, EC20, EC50, and H_2_O_2_).

## 3. Results

### 3.1. Implementation: H_2_O_2_ as Positive Control

No significant mortality of adult organisms was observed during H_2_O_2_ exposure (at least nine organisms were recorded per replicate).

**Figure 1:**
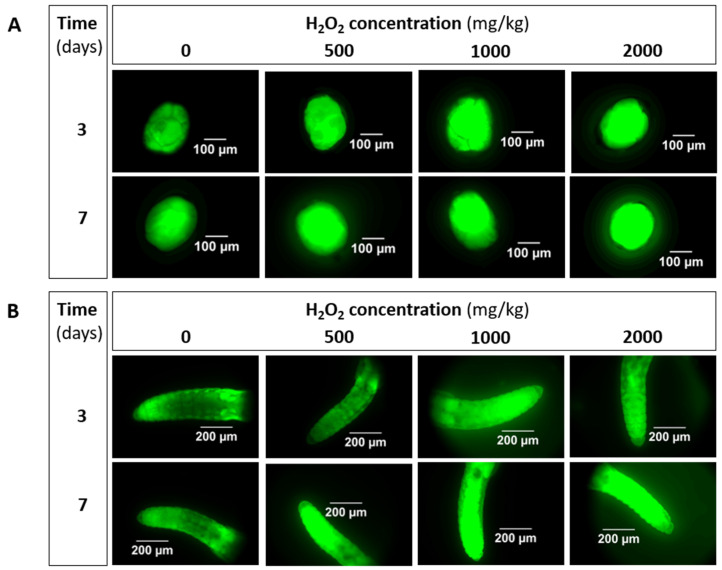
Microscopic images of ROS detection, illustrated by intensity of green fluorescence using DCFH-DA probe, in *Enchytraeus crypticus* (**A**) cocoons and (**B**) adults, when exposed to H_2_O_2_ in LUFA 2.2 soil, for 3 and 7 days.

Representative pictures of *E. crypticus* cocoons and adults exposed to H_2_O_2_ are shown in Figure 1.

**Figure 2:**
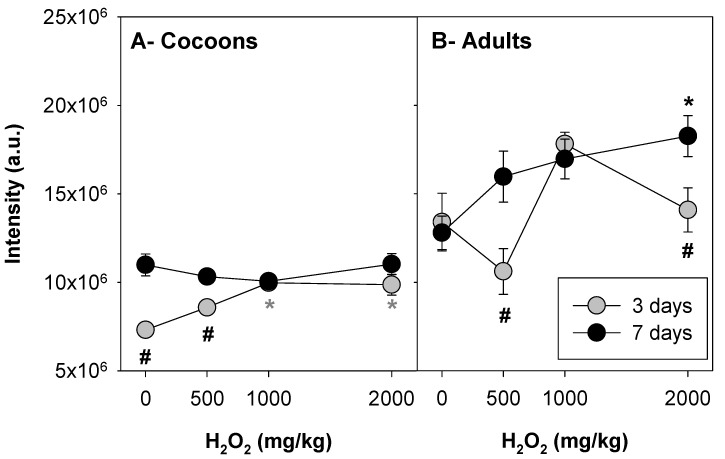
Fluorescence intensity (corrected total cell fluorescence, CTCF) determined in *Enchytraeus crypticus* (**A**) cocoons and (**B**) adults, when exposed to H_2_O_2_ in LUFA 2.2 soil, for 3 and 7 days. The results are expressed as average ± standard error (*n* = 12). a.u.: arbitrary units. *: *p* < 0.05 (Holm–Sidak test) in comparison with the respective control (un-spiked soil: 0 mg H_2_O_2_/kg). #: *p* < 0.05 (Holm–Sidak test) for 3 versus 7 days.

Inspection of the pictures (Figure 1) shows an increase in fluorescence intensity with increasing concentration, and an overall increase in intensity from 3 to 7 days of exposure. The results for corrected total cell fluorescence (CTCF) are shown in Figure 2.

The results show a significant interaction between time and H_2_O_2_ concentration (two-way ANOVA, see Supplementary Materials, for both cocoon and adults. For cocoons, in control conditions, the fluorescence intensity increased from 3 to 7 days, which was not the case for adults. For cocoons, 3 days of exposure to H_2_O_2_ caused a dose-dependent increase in the fluorescence levels, significantly different from control at 1000 and 2000 mg/kg, while after 7 days of exposure, the intensity levels were all higher, but similar (Figure 2A). For adults, there was a peak of intensity at 1000 mg/kg (although not significantly different from control) after 3 days of exposure, but after 7 days of exposure, there was a dose-dependent increase in intensity levels, significantly different from control at 2000 mg/kg (Figure 2B).

### 3.2. Ag NM300K Case Study

No significant mortality of adult organisms was observed during exposure to the several treatments, including Ag NM300K or H_2_O_2_ (at least eight organisms were recorded per replicate).

**Figure 3:**
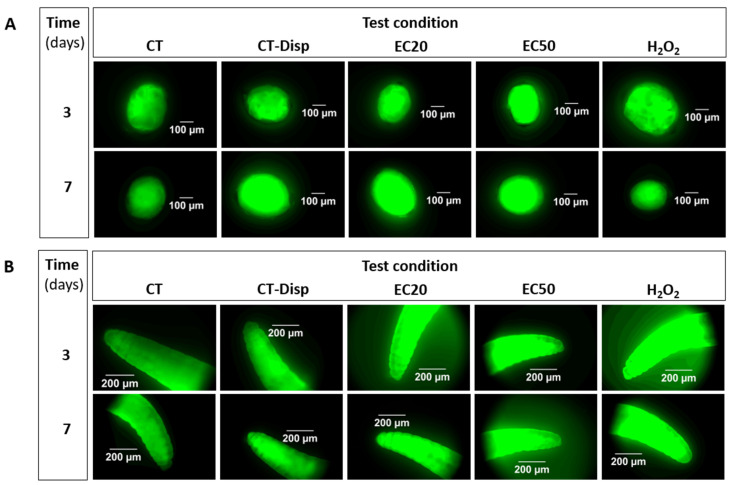
Microscopic images of ROS detection, illustrated by intensity of green fluorescence using DCFH-DA probe, in *Enchytraeus crypticus* (**A**) cocoons and (**B**) adults, when exposed in LUFA 2.2 soil, for 3 and 7 days, to different treatments (CT: control (un-spiked soil); CT-Disp: control-dispersant (dispersant: Tween 20 at equivalent to the highest concentration of Ag NM300K tested); EC20: Ag NM300K 20% effect concentration; EC50: Ag NM300K 50% effect concentration; H_2_O_2_: hydrogen peroxide as positive control (2000 mg/kg)).

Representative pictures of *E. crypticus* cocoons and adults exposed to Ag NM300K and controls (un-spiked soil: CT, control-dispersant: CT-Disp, and positive control: H_2_O_2_) are shown in Figure 3.

**Figure 4:**
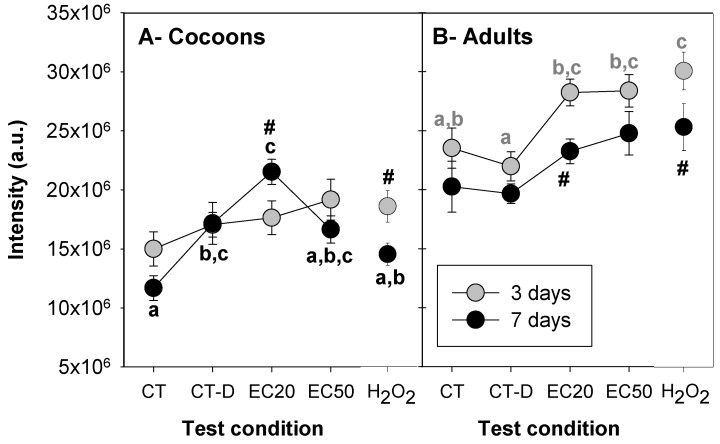
Fluorescence intensity (corrected total cell fluorescence, CTCF) determined in *Enchytraeus crypticus* (**A**) cocoons and (**B**) adults, when exposed in LUFA 2.2 soil, for 3 and 7 days, to different treatments (CT: control (un-spiked soil); CT-D: control-dispersant (dispersant: Tween 20 at equivalent to the highest concentration of Ag NM300K tested); EC20: Ag NM300K 20% effect concentration; EC50: Ag NM300K 50% effect concentration; H_2_O_2_: hydrogen peroxide as positive control (2000 mg/kg)). The results are expressed as average ± standard error (*n* = 12). a.u.: arbitrary units. Different letters represent statistically significant differences (*p* < 0.05, Holm–Sidak test) between test conditions, for each time. #: *p* < 0.05 (Holm–Sidak test) for 3 versus 7 days.

The inspection of the pictures indicates an increase in fluorescence associated with Ag NM300K treatments, for both *E. crypticus’* cocoons and adults. For cocoons exposed for 7 days to control-dispersant, there seems to be an increased intensity in comparison with the respective control (Figure 3A). The results for CTCF are shown in Figure 4.

Comparing the same treatments between developmental stages (cocoons and adults), the intensity levels were consistently higher in adults.

For cocoons, there was a significant interaction between time and test condition (two-way ANOVA, SI). Three days of exposure caused no significant differences in intensity between the test conditions. After 7 days, higher fluorescence intensity was detected for the EC20 of Ag NM300K, which was significantly different from the control (the lower fluorescence intensity detected), but not from the control-dispersant. The control and the control-dispersant were also significantly different from each other. There were no significant differences between EC50 of AgNM300K and both controls (water and dispersant). Comparing the two exposure times, Ag NM300K EC20 caused a significant increase in intensity levels from 3 to 7 days of exposure, while for H_2_O_2_ exposure, it decreased (Figure 4A).

For adults, there was no significant interaction between time and test condition, but there were differences within time and/or treatments (see SI for details). After 3 days of exposure, lower intensity levels were detected for the control-dispersant and control, and higher levels for H_2_O_2_ (significantly different from control). Both EC20 and EC50 of Ag NM300K caused a significant increase in intensity levels in comparison with the control-dispersant. After 7 days, there were no significant differences between the test conditions. Overall, the intensity levels decreased from 3 to 7 days of exposure, across treatments, significantly for Ag NM300K EC20 and H_2_O_2_.

## 4. Discussion

### 4.1. Implementation

A new method for ROS formation detection, based on the DCFH-DA method, was for the first time implemented in *E. crypticus* adults and also cocoons, i.e., during the embryonic stage.

Overall, in controls, cocoons produced lower intensity levels than adults. The detection of fluorescence depends not only on the presence of ROS (which oxidizes DCFH into the fluorescent DCF), but first of all on the capacity of DCFH-DA to enter the cells (a process that occurs by passive diffusion [27]). In the case of cocoons, the cocoon membrane might have acted as a barrier to the entrance of DCFH-DA, reducing its contact with *E. crypticus* embryos, hence reducing the fluorescence levels detected. This has been reported before, e.g., the fish egg membrane, the chorion, acts as a protective layer, showing low permeability to several compounds [28,29,30].

Cocoons’ ROS levels (as related to the levels of intensity, CTCF) increased in a dose-dependent way in the 3 days of exposure, but not after 7 days. This was probably related to a higher sensitivity to H_2_O_2_ of earlier developmental stages of the embryos (morula to the first invaginations at 4–5 days, and moving fusiform worms at days 8–9 [31]). The discussion on the sensitivity of animals’ different life stage to toxicants is not new. Our results are in agreement with those reported in the larva of *Scophthalmus maximus*, where yolk sac stages were more sensitive to Cd exposure than later larval stages [32], or in *Tisbe holothuriae*, whose nauplii were more sensitive to both Cd and Cu at 1 day of age compared with 5 days of age [33].

In adults, 7 days of exposure to H_2_O_2_ induced a dose-dependent increase in ROS levels, whereas at 3 of days exposure, the pattern was not clear. The oxidative stress and damage response is known to vary with time and concentration. For instance, in *E. albidus* exposed to zinc, lipid peroxidation (LPO) levels (a measure of oxidative damage to cell membranes) increased by day 4, but not after 8 days [6]; for Cd, higher LPO levels were observed at intermediate concentrations (5 mg Cd/kg) [6]. On the other hand, a dose-dependent increase in LPO was caused by Cu in *E. albidus* [7] and by Ag (nano and ions) in *Eisenia fetida* [4]. The variation observed here at 3 days of exposure might reflect the activation of anti-oxidant defence mechanisms, as an attempt to deal with the oxidative stress caused by H_2_O_2_, although not enough activation to prevent the dose/response accumulation of ROS that could have been measured after 7 days of exposure.

### 4.2. Ag NM300K Case Study

Exposure to Ag NM300K caused an increase in ROS formation, in *E. crypticus* adults and cocoons, as detected using the DCFH-DA method. However, the response varied with time in a non-linear way.

**Figure 5:**
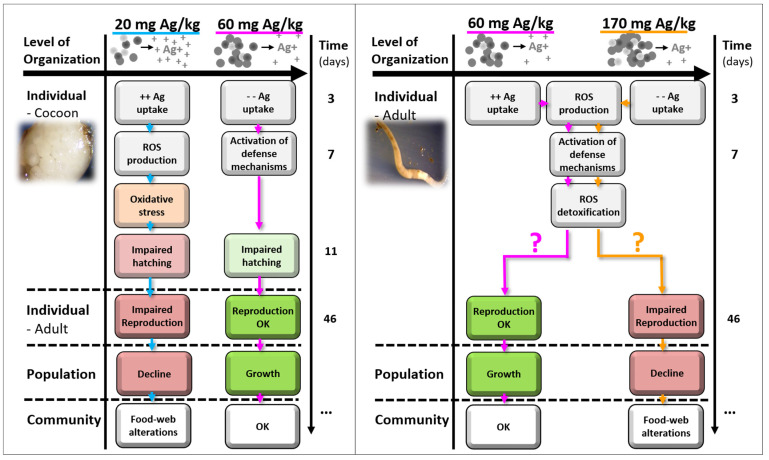
Adverse outcome pathway (AOP) for *Enchytraeus crypticus* cocoons (**left panel**) and adults (**right panel**) when exposed to Ag NM300K, in LUFA 2.2 soil, for 3 and 7 days, and linkage to adverse outcomes (as reported in Bicho et al. [21]).

For cocoons, despite the tendency for fluorescence intensity to decrease from 3 to 7 days, differences among treatments were detected after 7 days of exposure. Higher ROS levels were detected for Ag NM300K at the EC20, which aligns with the findings reported at organism level [21], where hatching was more affected at 20 mg Ag/kg than at 60 mg Ag/kg. Thus, ROS accumulation with consequent oxidative damage could be one of the sources for the higher effects on hatching reported at 20 mg Ag/kg, in comparison with 60 mg Ag/kg [21]. This non-monotonic dose–response was reported before for other NMs, at both organism level [21,34] and cellular level, e.g., in adult *E. crypticus* using DNA damage [20] or oxidative stress biomarkers [8] as endpoints. This may be explained by a more pronounced NM aggregation/agglomeration at higher concentrations [21]. Hence, we hypothesize that, at 20 mg Ag/kg, *E. crypticus* cocoons were exposed to more individual Ag particles and ions (rather than agglomerates), resulting in higher effects—ROS production (see Figure 5 for an illustration). Further, single particles are more likely to cross the cocoon membrane than the NMs’ aggregates, and will also oxidize faster, thus causing greater damage. Another hypothesis is that the higher concentration (EC50) activated the antioxidant defence mechanisms earlier, hence the embryos responded to the stress induced by Ag NM300K exposure and reduced the ROS. Accordingly, as reported in medaka (fish) embryos, the activation of superoxide dismutase (an antioxidant enzyme) for the highest concentrations of Ag NPs, in the first days of exposure, might have contributed to the reduction in ROS production observed at longer exposure periods [35]. The differences related to the different embryonic developmental processes were also highlighted by Wu and Zhou [35], who concluded that well-developed pre-hatching medaka are more resistant to oxidative stress caused by Ag NPs’ exposure than early embryonic stages. In our study, differences among treatments were detected after 7 days of exposure (and not after 3 days), indicating that this would be a better exposure time frame to implement the DCFH-DA method in *E. crypticus* cocoons. However, for H_2_O_2_, the opposite occurred. As the overall results indicate chemical-specific responses, we recommend to include both 3 and 7 days of exposure as the best option.

For adults, there was an overall decrease in fluorescence with time, indicating that the peak of ROS production occurred after 3 days (with significantly higher ROS levels for Ag NM300K treatments and H_2_O_2_ positive control). Previous short-term exposure studies using *E. crypticus* showed that exposure to Ag NM300K (similar doses and times) caused lipid peroxidation [8] and DNA damage [20] after 7 days of exposure, but not after 3 days. These results are in line with our current findings, showing that ROS peaking preceded the occurrence of damage at cellular level (lipid peroxidation and DNA damage). At gene level, 3 days of exposure to Ag NM300K EC20 and EC50 induce a similar transcriptional response, while it peaked after 7 days for the EC50 [19]. This indicates, as also shown at gene and cell levels, that oxidative stress and damage, as triggered by both ECx via short-term exposures, is a key initiating event for toxicity of Ag NM300K, while other mechanisms (or latter responses) are involved in the differentiation between EC20 and EC50 at organism level.

The fact that the response is time-specific, as observed here, is well-known, and the response to chemicals/materials depends on how fast those interact (i.e., react, dissolve, and so on) with the surrounding media (test media, extra- and intra-cellular space) and, consequently, with biota. For Ag NMs in particular, it was shown that, while short-term (molecular, cellular) responses are able to capture the differences between the nano and the ionic forms, often related to different uptake/internalization processes [36,37], at longer-term exposure times, toxicity tends to converge [38].

In contrast to cocoons’ response, for adults, higher ROS levels and discrimination of test treatments were obtained after 3 days of exposure. However, that was not the case when testing a concentration range of H_2_O_2_, thus reinforcing the chemical-specific time for response. Hence, we recommend to include both 3 and 7 days of exposure as the best design.

The observed differences between cocoons and adults exposed to Ag NM300K are likely related to different uptake routes. For cocoons, the membrane provides additional protection, not present in adults, that prevents the entry of materials. A previous study, based on histological analysis, indicates that exposure to Ag NM300K did not affect *E. crypticus* cocoon membrane integrity [39]. Further, although the cocoon membrane pore size is not known, the evidence is that it is smaller than the tegument of the juvenile/adult worm (see [39]). Hence, after leaving the cocoon, animals are expected to be less protected and more exposed via tegument (e.g., Ag entry and ROS formation). For adults, besides dermal intake, oral uptake is possible and likely to occur. The size of the mouth part in an adult *E. crypticus* is in the size range of 100 μm [23] and individual Ag particles are 15 nm in size. This entry source can explain the higher initial uptake of Ag in adults, and hence faster effects.

A schematic adverse outcome pathway (AOP) format representation was drafted for the main results and hypothesis raised for the impact of Ag (NM300K) in *E. crypticus* when exposed from cocoons and from adult stages (Figure 5). The adverse outcomes, at individual, population, and community levels, are derived and discussed in Bicho et al. [21] in further detail.

Overall, both cocoons and adults are suitable models to detect ROS using the DCFH-DA method and fluorescence microscopy. Among the potential advantages of this method is the use of the whole animal/cocoon, without the need for tissue homogenization (although the method can be applied to tissue homogenate as described in Shao et al. [14]). The visualization of the whole animal (possible for smaller organisms) allows the eventual identification of differences between tissues, and the localization, at the tissue and organ level, where the main response is taking place.

## 5. Conclusions

ROS detection using the DCFH-DA method was successfully implemented in *E. crypticus* adults and cocoons for the first time. Exposure to Ag NM300K induced ROS formation in both cocoons and adult organisms. Cocoons showed lower levels of fluorescence compared with adults, possibly related to the low permeability of the cocoon membrane. The ROS levels in cocoons exposed to Ag NM300K (higher for the EC20 than for the EC50) align with the results reported at organism level (non-monotonic response). In adults, an increase in ROS levels at 3 days seems to precede oxidative damage, as reported to occur later (7 days) using a similar test design.

Overall, the DCFH-DA method proved to be effective and can be used as a novel tool to detect ROS formation in *E. crypticus* after short-term exposure to chemicals, including nanomaterials. Given the chemical-specific time to induce cellular response, we recommend the use of more than one exposure period (i.e., 3 and 7 days) for the assessment.

## Figures and Tables

**Table 1:** Summary of the experimental design and tested concentrations, for *Enchytraeus crypticus* adults (mature, well-developed clitellum) and cocoons (1–2 days post-laying), exposed in LUFA 2.2 soil to hydrogen peroxide (mg H_2_O_2_/kg soil) and Ag NM300K (mg Ag/kg soil). disp.: control dispersant; ^#^ dispersant: Tween 20 equivalent to the highest concentration of Ag NM300K tested; * reproduction effect concentrations (EC20 and EC50) (Bicho et al. [21]); ^§^ hatching effect concentrations (EC20 and EC50) (Bicho et al. [21]).

#	Test Level	Test Substance/Material	*E. crypticus* Development Stage	Concentrations (mg/kg)	Exposure Time (Days)
1	implementation	H_2_O_2_	adults	0, 500, 1000, 2000	3, 7
2			cocoons		
3	case study	Ag NM300K	adults	disp.^#^, 60 *, 170 *	3, 7
4			cocoons	disp.^#^, 20 ^§^, 60 ^§^	
5		H_2_O_2_ (positive control)	adults	0, 2000	3, 7
6			cocoons		

## Data Availability

The data presented in this study are available on request from the corresponding author.

## Supplementary Materials

The following are available online at https://www.mdpi.com/article/10.3390/toxics9100232/s1, Tables S1–S4: Two-way ANOVA results.
