# Supplementary material for: Reactive Oxygen Species Detection Using Fluorescence in Enchytraeus crypticus—Method Implementation through Ag NM300K Case Study
Source: Toxics. 2021 Sep 24;9(10):232. doi: 10.3390/toxics9100232 (PMC8541345; doi:10.3390/toxics9100232)
Supplement: Supplementary file 1 [file toxics-09-00232-s001.zip › toxics-1374124 SM.pdf]

toxics MDPI

Supplementary Materials: Reactive Oxygen Species Detection Using Fluorescence in *Enchytraeus crypticus*—Method Implementation through Ag NM300K Case Study

Susana I.L. Gomes, Ana B. Neves, Janeck J. Scott-Fordsmand and Mónica J.B. Amorim

Table S1. to S4. Two-way ANOVA results.

Table S1. Two-way ANOVA results for *Enchytraeus crypticus* cocoons exposed to H_2O_2, in LUFA 2.2 soil for 3 and 7 days. Dependent variable: test 1) method optimization; CTCF cocoons. Independent variables (factors): time and conc. H2O2.

Source of Variation	DF	SS	MS	F	*p*
time	1	6.67×10^{13}	6.67×10^{13}	24.404	<0.001
conc. H2O2	3	2.43×10^{13}	8.10×10^{12}	2.96	0.037
time x conc. H2O2	3	4.08×10^{13}	1.36×10^{13}	4.976	0.003
Residual	88	2.41×10^{14}	2.74×10^{12}		
Total	95	3.73×10^{14}	3.92×10^{12}		

Table S2. Two-way ANOVA results for *Enchytraeus crypticus* adults exposed to H_2O_2, in LUFA 2.2 for 3 and 7 days. Dependent variable: test 1) method optimization; CTCF adults. Independent variables (factors): time and conc. H2O2

Source of Variation	DF	SS	MS	F	*p*
time	1	9.79×10^{13}	9.79×10^{13}	5.481	0.021
conc. H2O2	3	3.27×10^{14}	1.09×10^{14}	6.109	<0.001
time x conc. H2O2	3	1.85×10^{14}	6.17×10^{13}	3.455	0.02
Residual	88	1.57×10^{15}	1.79×10^{13}		
Total	95	2.18×10^{15}	2.30×10^{13}		

Table S3. Two-way ANOVA results for *Enchytraeus crypticus* cocoons exposed to Ag NM300K, in LUFA 2.2 soil for 3 and 7 days. Dependent variable: test 2) method validation; CTCF cocoons. Independent variables (factors): time and test condition: CT: control; CT-Disp: control-dispersant; EC20: Ag NM300K 20% effect concentration; EC50: Ag NM300K 50% effect concentration; H2O2: Hydrogen Peroxide as positive control (2000 mg/kg).

Source of Variation	DF	SS	MS	F	*p*
Time	1	4.13×10^{16}	4.13×10^{16}	1956.00	0.165
Test condition	4	5.05×10^{17}	1.26×10^{17}	5968.00	<0.001
Time x Test condition	4	2.51×10^{17}	6.28×10^{16}	2973.00	0.022
Residual	110	2.32×10^{18}	2.11×10^{16}		
Total	119	3.12×10^{18}	2.62×10^{16}		

Table S4. Two-way ANOVA results for *Enchytraeus crypticus* adults exposed to Ag NM300K, in LUFA 2.2 soil for 3 and 7 days. Dependent variable: test 2) method validation; CTCF adults. Independent variables (factors): time and test condition: CT: control; CT-Disp: control-dispersant; EC20: Ag NM300K 20% effect concentration; EC50: Ag NM300K 50% effect concentration; H2O2: Hydrogen Peroxide as positive control (2000 mg/kg).

Source of Variation	DF	SS	MS	F	*p*
Time	1	4.31×10^{17}	4.31×10^{17}	15037.00	<0.001
Test condition	4	8.84×10^{17}	2.21×10^{17}	7701.00	<0.001

Time x Test condition	4	2.91×10^{16}	7.27×10^{15}	0.253	0.907
Residual	110	3.16×10^{18}	2.87×10^{16}		
Total	119	4.50×10^{18}	3.78×10^{16}		
